# Biogeography and Systematics of the Genus *Axyris* (Amaranthaceae s.l.)

**DOI:** 10.3390/plants11212873

**Published:** 2022-10-27

**Authors:** Alexander P. Sukhorukov, Valeriia D. Shiposha, Maria Kushunina, Maxim A. Zaika

**Affiliations:** 1 Department of Higher Plants, Biological Faculty, Lomonosov Moscow State University, 119234 Moscow, Russia; 2 Laboratory Herbarium (TK), Tomsk State University, Lenin Ave. 36, 634050 Tomsk, Russia; 3 Department of Plant Physiology, Biological Faculty, Lomonosov Moscow State University, 119234 Moscow, Russia

**Keywords:** Amaranthaceae, Asia, *Axyris*, biogeography, molecular phylogeny, reproductive characteristic

## Abstract

*Axyris* is a small genus of six species with a disjunct geographic range. Five species are present in Siberia, Central Asia, the Himalayas, and Tibet, whereas *Axyris caucasica* has been registered in the Central Caucasus only. *Axyris* species diversity is the highest in the Altai Mountains (four spp.), followed by the Tian Shan and Pamir Mountains (three spp.), and the Himalayas and Tibet (two spp.). *Axyris sphaerosperma*, sometimes considered endemic to Southern Siberia, in fact has a disjunct range: it is present in the lowlands of Eastern Siberia and in the Altai, Tian Shan, and Pamir Mountains. It has also been found in Mongolia and China for the first time. An updated detailed distribution of *Axyris* in Siberia is presented on the basis of thorough herbarium revisions. One nuclear and three plastid markers were selected for phylogenetic analysis. Divergence times were estimated using a time-calibrated Bayesian approach. *Axyris* shows two major clades: an *Axyris amaranthoides* clade and a clade including the remaining species. The latter clade consists of two subclades (*A. sphaerosperma*/*A. caucasica* and *A. mira*/*A. prostrata* + *A. hybrida*). The crown age for *Axyris* dates back to the Early Pliocene (~5.11 mya, the Zanclean). The ancestral range of *Axyris* covers Southern Siberia, Mongolia, NW China, and the Tian Shan/Pamir Mountains, with extensions toward Eastern Siberia, the Himalayas/Tibet, and the Caucasus. Fruit and seed characteristics of *Axyris* are discussed with reference to the present phylogenetic results. Closely related *A. sphaerosperma* and *A. caucasica* have the thickest seed coat among all Chenopodiaceae, and these traits have probably evolved as adaptations to extremely low winter temperatures. This reproductive peculiarity may explain the disjunct range of *A. sphaerosperma*, which is restricted to harsh climatic conditions.

## 1. Introduction

*Axyris* L. is a small genus of Chenopodiaceae Vent. s.str. (Amaranthaceae Juss. s.l.) comprising six species [1]. All of them share an annual life form, stellate pubescence of the stem and leaves, unisexual flowers, and one-seeded indehiscent fruits, usually with ear-like outgrowths in their upper part originating from the pericarp [2]. Although the genus is easily recognizable in the field, identification at the species level has often led to confusion due to the absence of reliable diagnostic characteristics. Three species—*Axyris amaranthoides* L., *A. hybrida* L., and *A. sphaerosperma* Fisch. & C.A.Mey.—have often been misidentified owing to their upright stems, similar leaf shapes, and overlapping geographic ranges in some parts of temperate Asia. Diagnostic methods for each species were greatly improved only recently, and pubescence details of stems and leaves and specific features of fruits and seeds appear to be the key discriminatory traits [1,2]. All *Axyris* species produce both heterocarpous and heterospermous diaspores, and morphoanatomical traits of fruit types are now regarded as diagnostic at the specific level [1,2].

The main center of distribution of *Axyris* lies in Central Asia [3,4,5,6] and the Himalayas/Tibet [1]. A single species, *Axyris caucasica* (Somm. & Lev.) Lipsky, is present in the Greater Caucasus [3]. *Axyris* therefore demonstrates substantial geographical disjunction between two parts of its native range: temperate East Asia and the Greater Caucasus. Among all *Axyris* species, only *A. amaranthoides* has become an adventive alien with a scattered distribution in the Russian Far East [7], Eastern Siberia (Sakha Rep.; [8]), Eastern Europe [9], Fennoscandia [10], the North Caucasus [11], and North America [12].

*Axyris* is the type genus of the tribe Axyrideae G.Kadereit & Sukhor. [13]. The genus seems to be monophyletic, judging by two or three species included in a phylogenetic analysis [13,14,15], but infrageneric relationships have not been elaborated so far. The aims of the present study were (i) to reveal the main diversity centers of *Axyris*, (ii) to construct an *Axyris* phylogeny comprising all the species of the genus and to revise the systematics of the genus, and (iii) to trace biogeographical history and radiation of *Axyris* on the basis of the phylogenetic analysis.

## 2. Results

### 2.1. Species Distribution of Axyris in Asia

**Figure 1:**
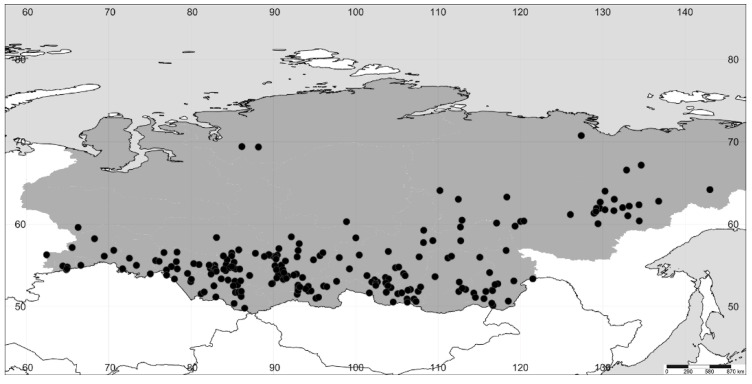
The distribution of *A. amaranthoides* in Siberia (the region itself is colored gray).

**Figure 2:**
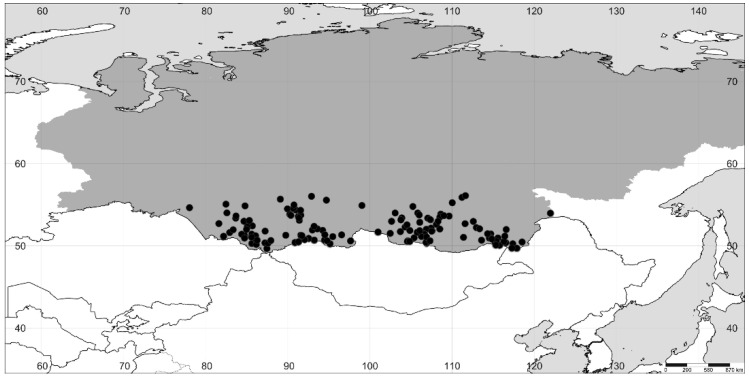
The distribution of *A. hybrida* in Siberia (the region itself is highlighted in gray).

**Figure 3:**
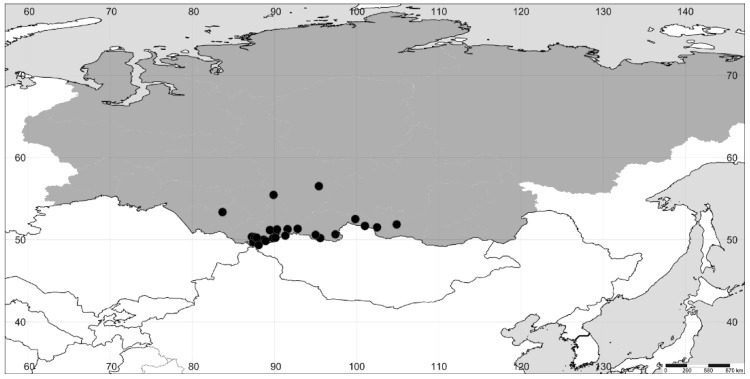
The distribution of *A. prostrata* in Siberia (the region itself is colored gray).

**Figure 4:**
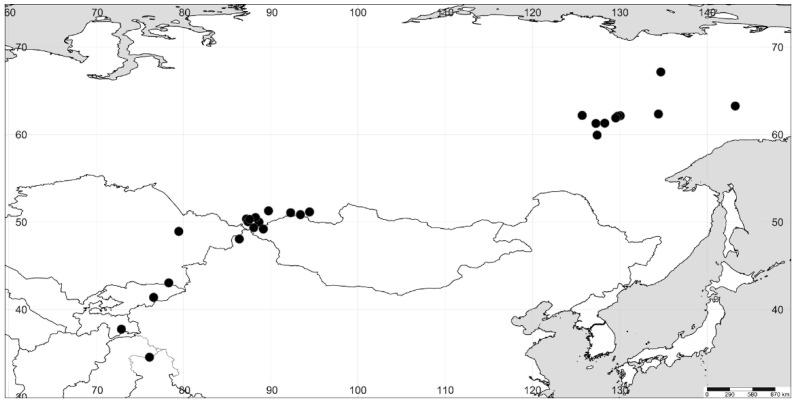
The distribution of *A. sphaerosperma*.

**Figure 5:**
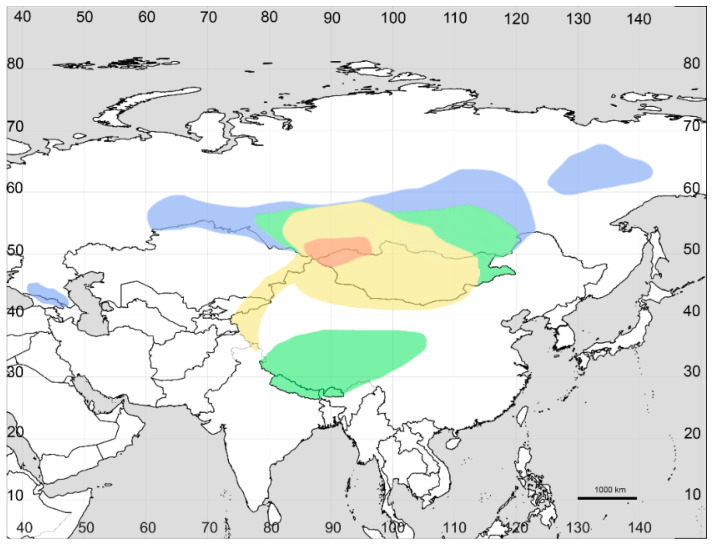
Species diversity of *Axyris*. Colored areas: orange, four species; yellow, three species; green, two species; blue, one species.

We revised the distribution of *Axyris* in Siberia, and in particular, we found that the range of *A. sphaerosperma* is drastically different from the one previously reported (Figure 1, Figure 2, Figure 3 and Figure 4). Based on our recent results and the previous study [1], the highest species diversity of *Axyris* is seen in Southern Siberia, Mongolia, and NW China (Figure 5), where four species are present (*A. amaranthoides, A. hybrida, A. prostrata,* and *A. sphaerosperma*), followed by the Tian Shan and Pamir Mountains (*A. hybrida, A. prostrata*, and *A. sphaerosperma*) and the Himalayas/Tibet (*A. mira* and *A. prostrata*). One native species each is present in Eastern Siberia (Sakha [Yakutiya] Republic) and in the Greater Caucasus: *A. sphaerosperma* and *A. caucasica*, respectively.

### 2.2. Dated Molecular Phylogeny of Axyris

**Figure 6:**
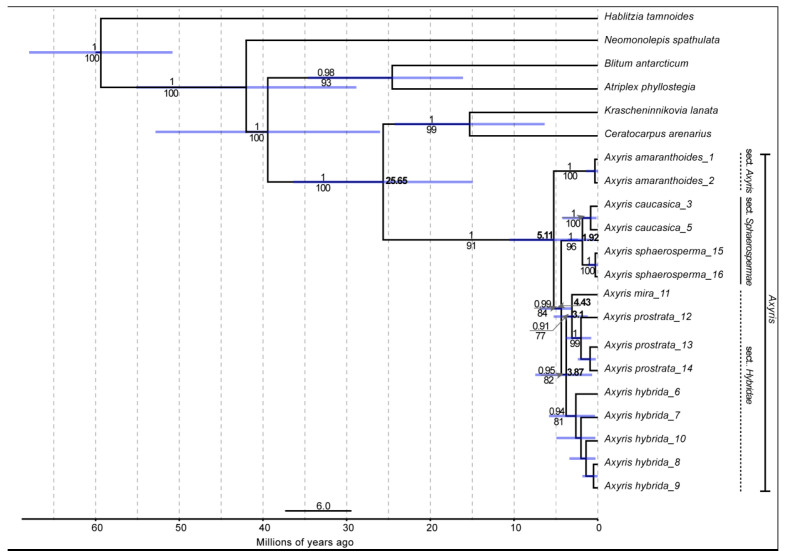
The maximum clade credibility tree of *Axyris* obtained by the BEAST2 analysis was subjected to secondary calibrations (see Methods). Posterior probabilities resulting from the Bayesian analysis are indicated above branches (only values ≥ 0.9), and the numbers below branches refer to bootstrap values resulting from the ML analysis (only values ≥ 70). Mean divergence times (values at some nodes) are shown with their 95% HPD (blue bars).

The combined dataset of all four markers (ITS, *rbcL* coding gene, *atpB*-*rbcL* and *trnL*-*F* intergenic spacers) comprises 2588 aligned bp and 21 accessions. ML and Bayesian analyses revealed identical topologies. *Axyris* is monophyletic with high support and it is recovered as a sister clade to *Krascheninnikovia* and *Ceratocarpus* (Figure 6; BSL 100; PP 1). *Axyris amaranthoides* is resolved as sister to the rest of the species (BSL 91; PP 1). *Axyris caucasica* and *A. sphaerosperma* form a clade (BSL 96; PP 1) sister to a well-supported clade (BSL 82; PP 0.95) consisting of *A. mira*/*A. prostrata* + *A. hybrida*.

The diversification of *Axyris* started in the late Eocene ca. 25.65 mya (stem age, 95% HPD: 36.3–14.97). The crown age of *Axyris* dates back to ca. 5.11 mya (95% HPD: 10.91–2.82 mya) which suggests that the genus originated during the early Pliocene (the Zanclean stage).

### 2.3. Biogeographic Analyses and Radiation of Axyris

**Figure 7:**
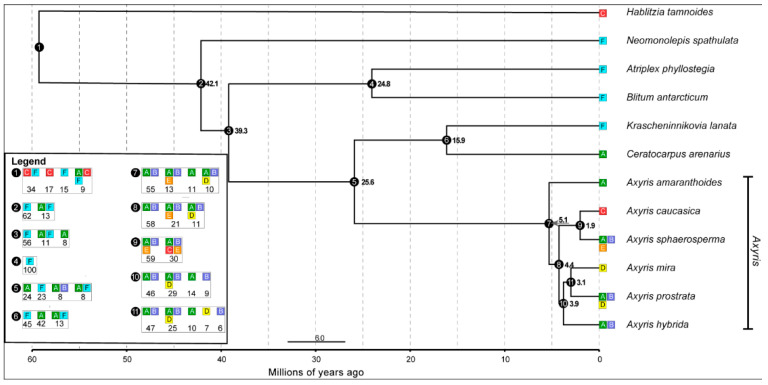
The time-calibrated tree of six *Axyris* taxa, as generated in BEAST2 allowing one accession per species. The ancestral area analysis was conducted by means of BioGeoBEARS in R v.3.3.2. Coding of biogeographical areas: A: Southern Siberia, Mongolia, and NW China; B: Tian-Shan and Pamir; C: Greater Caucasus; D: Himalayas/Tibet; E: Eastern Siberia (Sakha Rep.); F: North America.

The ancestral area for the crown node of *Axyris* could be estimated for the regions A (Southern Siberia, Mongolia, NW China) and B (Tian Shan, Pamir Mountains) (AB: *p* = 0.55, ABE: *p* = 0.13, A: *p* = 0.11; ABD: *p* = 0.10; Figure 7). It is likely that the ancestral *Axyris* domain can be linked to the geographic area where four species of the genus are present today (Figure 5; marked with orange). For all other nodes (except node 9, ABE: *p* = 59, ABCE: *p* = 30), the likely ancestral domain is also confined to AB areas with varying probabilities (Figure 7, see Legend). Further dispersal of *Axyris* proceeded towards the Himalayas/Tibet (Region D: *A. mira* and *A. prostrata*), which was colonized once, and then Eastern Siberia (Sakha Republic, Region E) (*A. sphaerosperma*) and the Caucasus, Region C (*A. caucasica*).

## 3. Discussion

### 3.1. The Distribution of Axyris in Siberia

Four out of six *Axyris* species—namely *A. amaranthoides*, *A. hybrida*, *A. prostrata*, and *A. sphaerosperma*—are native to Siberia [3]. According to the latest examination of the genus in Siberia [16], *A. amaranthoides* is widespread in Southern Siberia, with a few records in Northern and Eastern Siberia; *A. hybrida* mostly occurs in Southern Siberia with scattered findings in Eastern Siberia; and *A. prostrata* is present in Southern Siberia. No distribution map of *Axyris sphaerosperma* was provided by M. Lomonosova [16], but several findings in Altai and Tyva Republics were reported, as was one finding in the city of Yakutsk (Sakha Republic). 

According to our investigations, *A. amaranthoides* is a common plant in most of Siberia, but its records in the northern and northeastern parts of the region represent recent migrations as a consequence of human activities. In Eastern Siberia, it is more frequently found in settlements and along main transport routes, especially in the Lena River basin. *A. hybrida* occurs across Southern Siberia, mostly in the Altai and Sayan Mountains but also in the adjacent lowlands. The findings of *A. hybrida* in Sakha Republic (Eastern Siberia) reported by the authors of refs. [16] and [8] are not confirmed, and these records in fact belong to *A. amaranthoides*. As stated by M. Lomonosova [16] and confirmed by us, the geographic range of *A. prostrata* is mostly confined to the Altai and Sayan Mountains, with scattered records in the uplands of Baikalia and Krasnoyarsk Krai.

### 3.2. A Geographical Puzzle concerning A. sphaerosperma

Out of all *Axyris* species, only *A. sphaerosperma* possesses a disjunct range, with two fragments (Figure 4) reported here for the first time. The first (main) fragment is located in the Altai and Sayan Mountains, with extensions to the Tian Shan, Pamir, and North Himalayas [1,3,5]. Here we also add the first but expected records for Mongolia (Mongolian Altai, Bayan-Ulgii prov., Tavan-Bogd Mts., Tsagaan-Gol River basin, 4 August 2001, I. Krasnoborov, A. Shmakov, and D. German 82 (NS0009424); and China (Irenchabirga, 9000 ft, 10 September 1879, A. Regel [LE]; Zaisan expedition [Xinjiang prov., Habahe county], Kobuk River basin, 20 July 1914, V. Sapozhnikov s.n., all as *A. amaranthoides* [LE]) that have not been mentioned earlier (e.g., [6,17,18]). These new findings were made close to those in Altai Republic (Southern Siberia, Russia). In the main fragment of its range, *A. sphaerosperma* occurs at high altitudes, between 1500 and 3000 m a.s.l. [19]. The second fragment is located in the lowlands of Eastern Siberia (Sakha Republic). Both areas are characterized by extremely low winter temperatures and a short vegetation period lasting from mid-May to mid-September. The reasons for such a fragmentary range of *A. sphaerosperma* are unclear. Nonetheless, it should be noted that *A. sphaerosperma* is well adapted to the harsh climatic conditions by forming a persistent soil seed bank. Although all *Axyris* species have heteromorphic fruits and seeds with different longevity, some fruits of *A. sphaerosperma* and *A. caucasica* have sclereids and contain seeds with a very hard and thick (up to 100–115 μm) seed coat [2]. Such thickness of the seed coat is exceptional for Chenopodiaceae [9,20] and is a good example of physical dormancy in species growing under harsh climatic conditions.

### 3.3. The Origin of Axyris

The origin of this genus is probably connected with the orographic and climatic changes in the late Miocene/early Pliocene, which are manifested in renewed tectonism and further aridification of the lowlands in South Siberia and Tian-Shan [21]. The same region of origin, for example, is reported for *Krascheninnikovia ceratoides* (L.) Gueldenst. [22,23], a species also belonging to Axyrideae [13]. It is still not clear whether the origin of *Axyris* and *Krascheninnikovia* is linked to high-altitude or lowland steppes, but it is thought that the late Miocene and early Pliocene are the time scales where a continent-wide restructuring of the distribution of landscape-forming elements was taking place, including a new zonal structure component: steppe formation [24]. Our time-calibrated tree coincides with previous study [25]. Divergence time between *Axyris* and *Krascheninnikovia* + *Ceratocarpus* group is 26.5 mya and between *Krascheninnikovia* and *Ceratocarpus* is 16.4 mya, which generally corresponds with our data (25.6 and 15.9 mya, respectively). 

According to the altitudinal gradient, species of *Axyris* can be subdivided into two main groups: (1) predominantly lowland species (only *A. amaranthoides*) and (2) predominantly mountain species (all the other taxa), sometimes penetrating onto the lowlands (*A. hybrida*, *A*. *prostrata*, and *A. sphaerosperma*). None of them can be classified as desert plants, and there is evidently a gap in the distribution of the genus in the Taklamakan desert. A considerable gap between the main distribution area of the genus located in temperate East Asia and a small fragment in the Greater Caucasus (*A. caucasica*) can be explained by climatic changes, including Paleo-Caspian Transgression in the lowlands of Kazakhstan during the Pliocene and Pleistocene [26]. As stated earlier [1], one type of reproductive diaspores of both *A. sphaerosperma* and *A. caucasica* is very thick and provides high seed longevity. The reproductive strategy of these species is connected with the adaptation to extremely low winter temperatures. We suppose that the precursors of these species were present in the Pliocene and early Pleistocene in the lowlands of the Aralo-Caspian floristic province reaching the Caucasus, the areas where permafrost never disappeared during warm phases [27]. Decreasing permafrost led to the desertification of the landscape [28] and therefore could induce the disappearance of cold-adapted species in the lowlands and their isolation in the areas with much colder winter temperatures. 

### 3.4. Systematics of Axyris

This topic is elaborated based on a molecular phylogeny for the first time here. As proposed earlier [1,2], carpological data fully support the new systematic subdivision of *Axyris* into three sections.

- Gen. Axyris L., Sp. Pl.: 979 (1753).

Monoecious annuals covered with stellate hairs sometimes intermixed with simple multicellular hairs. Leaves short- or long-petiolate; blades ovate, oblong, spatulate, or lanceolate, entire, rarely undulate. Male flowers arranged in terminal spike-like inflorescences up to 8 cm long, with minute perianths of five free hyaline segments and with 2–5 stamens; female flowers located in bract axils, with five prominent hyaline perianth segments (two of which are erroneously called bracteoles). Fruits always dimorphic (heterocarpous); pericarp tightly adhering to the seed coat, usually with ear-like appendages at the apex of the fruit. Seeds also dimorphic (with thick and thin testal layer of the seed coat). Embryo vertical, horseshoe-shaped (in flattened fruits), or annular (in spheroidal fruits); perisperm present.

Six species in Eurasia, predominantly in Central Asia; one (*A. amaranthoides*) grows as an alien in many parts of Europe and North America. 

Lectotype (Jonsell and Jarvis in [29]): *Axyris amaranthoides* L.

#### The New Sectional Subdivision of the Genus

- Key to the sections

1. Stellate hairs with short and long rays; black fruits spheroidal, brown fruits compressed—*Axyris* sect. *Sphaerospermae*
  - Stellate hairs with short rays; fruits of both types compressed … 2
2. Ear-like pericarp appendages touching each other; fruits without concentric ridges—*Axyris* sect. *Axyris*
  - Ear-like pericarp appendages not touching each other; black fruits with concentric ridges—*Axyris* sect. *Hybridae*

***Axyris* sect. *Axyris***

Stellate hairs with short or slightly elongated rays; fruits of both types compressed; black fruits smooth (without concentric sculpture) with small apical appendages touching each other, seed coat 30–45 (55) μm; brown fruits with large ear-like appendages also touching each other, seed coat 20–25 μm.

One species, *Axyris amaranthoides* L. (type species of the genus).

**1.**  ***Axyris amaranthoides*** L., Sp. Pl.: 979 (1753).

Lectotype (Jonsell and Jarvis in [29]): Herb. Linn. 1101.4 (LINN!). Image available at: http://linnean-online.org/10713/ (accessed on 2 June 2022)

= *Axyris prostrata* L. var. *diffusa* Fenzl in Ledeb., Fl. Ross. 3: 714 (1849).

Lectotype (designated here): “herb. Ledebour” (LE01019599!) upper twig at fruiting stage.

= *Axyris amaranthoides* L. var. *dentata* A.I.Baranov, Zap. Kharbin. Obsch. Estestvoisp. Etnogr. 12: 35 (1954).

≡ *Axyris amaranthoides* L. f. *dentata* (A.I.Baranov) Kitag., Neolin. Fl. Manshur.: 248 (1979). 

Type: [China] prov. Cheilungkiang [Heilongjang prov.] ad ostium fl. Argun, pagus Strelka, in ruderatis, 29 July 1950, A. Baranov [type collection not designated]. 

Note. A variety with slightly undulate leaves. Such plants are sometimes found in different parts of the species range.

= *Axyris koreana* Nakai, J. Jap. Bot. 15(9): 525 (1939). 

Type: Korea, prov. Kannan, secus lacum artificialem tractus Tyôsin, 7 August 1938, S. Zen *5* (TI—image seen!). 

***Axyris* sect. *Hybridae* Sukhor., sect. nov.**

Stellate hairs with short or slightly elongated (*A. prostrata*) rays; fruits of both types compressed; black fruits with concentric ridges or rugose, with small apical appendages not touching each other, seed coat 25–50(65) μm; brown fruits with small or hardly noticeable appendages also not touching each other, seed coat 7–15 μm.

Type species: *Axyris hybrida* L.

Three species: *Axyris hybrida* L., *A. prostrata* L., and *A. mira* Sukhor., in Central Asia, Southern Siberia, and the Himalayas/Tibet. Two of them are present in Russia.

**2.**  ***Axyris hybrida*** L., Sp. Pl.: 980 (1753).

Lectotype (Sukhorukov, Fedd. Repert. 116(3–4): 175 (2005)): Herb. Linn. 1101.5 (LINN!). 

= *Axyris amaranthoides* L. var. *stricta* Fenzl in Ledeb., Fl. Ross. 3: 713 (1851).

Lectotype (designated here): “Herb. Ledebour 8711” LE!, left-handed specimen).

= *Axyris hybrida* L. var. *eravinensis* Peshkova in Malyshev and Peshkova, Fl. Tsentrl. Sibiri 1: 299 (1979).

Type: [Russia] Buryat Republic, Eravninsky distr., in loco dicto Barun-Uldurga, ad viam, 23 July 1953, Takhistova s.n. (NSK—image seen!).

**3.**  ***Axyris prostrata*** L., Sp. Pl.: 980 (1753).

Lectotype (Sukhorukov, Fedd. Repert. 116(3–4): 175 (2005)): Herb. Linn. 1101.6 (LINN!). 

= *Axyris prostrata* L. var. *latifolia* Fenzl in Ledeb., Fl. Ross. 3: 714 (1849).

Lectotype (designated here): “Altai. 1826 [fr.], № 871.2.2. Herb. Ledebour”. (LE01019600!).

= *Axyris pamirica* B.Fedsch., Acta Hort. Petrop. 24(3): 342 (1905).

≡ *Axyris prostrata* L. var *pamirica* B.Fedtsch., Acta Hort. Petrop. 24(3): 342 (1905).

Holotype: [Tajikistan, Gorny Badakhshan] Pamir, Gorumdy, 27 August 1904, B.A. Fedchenko s.n. (LE!).

**4.**  ***Axyris mira*** Sukhor., Willdenowia 41(1): 76 (2011).

Holotype: [India, Uttarakhand State] Kumaon, Milam glacier, 12,500 ft above the sea, 28 August 1848, R. Strachey and J.E. Winterbottom 2 (LE!).

***Axyris* sect. *Sphaerospermae* Sukhor., sect. nov.**

Stellate hairs with short and long rays; black fruits spheroidal, brown fruits compressed; black fruits smooth or with ± longitudinally striate sculpture (concentric ridges absent), apical appendages small or almost unnoticeable, not touching each other, seed coat (40)50–90(115) μm; brown fruits with small or hardly noticeable appendages also not touching each other, seed coat 12–20(25) μm.

Type species: *Axyris sphaerosperma* Fisch. & C.A.Mey.

Two species: *Axyris sphaerosperma* (in Central Asia, Sakha Republic, Pamir/Tian Shan Mts., and North Himalayas) and *A. caucasica* (Somm. & Lev.) Lipsky (in the Greater Caucasus).

**5.**  ***Axyris sphaerosperma*** Fisch. & C.A.Mey. in Fisch., C.A.Mey. & Ave-Lall., Index Sem. Hort. Petrop. 6: 46 (1839).

Lectotype (Sukhorukov, Fedd. Repert. 116(3–4): 175 (2005)): [Russia, Altai Republic] In regione orientali fl. Altaicae, Tschuja. [anno] 1839, [anonymous] (LE01019601!).

**6.**  ***Axyris caucasica*** (Somm. & Lev.) Lipsky, Fl. Cauc.: 430 (1899).

≡ *Axyris sphaerosperma* Fisch. & C.A.Mey. var. *caucasica* Somm. & Lev., Acta Hort. Petrop. 13(2): 196 (1894).

Lectotype (designated here): [Russia, North Caucasus, Karachay-Cherkessia Rep.] in jugo Tieberdinski pereval dicto, inter flumina Tieberda et Do-ut [Dout], ditionis Kuban, 1400 m, 1 September 1890, S. Sommier and E. Levier s.n. (LE!).

## 4. Materials and Methods

The taxonomic revision of the herbarium material was conducted at ABGI, G, LE, LECB, MHA, MSK, MSKU, MW, MWG, MOSP, NS, NSK, PE, PVB, RV, RWBG, TK, TLT, VOR, and WIR. The field investigations were carried out by the first author (A.P.S.) in Southern Siberia and the Far East (Amur Oblast). The data on the distribution of *A. mira* and *A. prostrata* in Himalaya and Tibet were taken from ref. [1]. All records for each species are given in Appendix A. Distribution maps are based on the specimens cited and were prepared using the SimpleMappr online tool (http://www.simplemappr.net, accessed on 4 May 2022). 

### 4.1. Sampling and DNA Extraction, Amplification, and Sequencing

Sixty accession numbers were included in the phylogenetic analyses, representing all six *Axyris* species, as well as 24 accession numbers as outgroups from Chenopodiaceae/Amaranthaceae; the samples are listed in Table 1.

DNA was extracted from 5–10 mg of dried leaf samples from the herbarium specimens by means of the DNeasy Plant Mini Kit (Qiagen, Valencia, CA, USA). One nuclear (the nuclear ribosomal internal transcribed spacer, nrITS) and three plastid markers (protein-coding gene *rbcL* and two intergenic spacers: *atpB*-*rbcL* and *trnL*-*trnF*) were selected for the phylogenetic analysis. 

PCRs were carried out in Thermal Cycler T100 (Bio-Rad, USA) using the primers and cycler programs listed in Table 2.

PCRs for all primers were conducted in 25 μL reaction mixtures consisting of 5 μL of DNA (10 ng/μL), 1 μL of each primer, 0.5 μL of Encyclo polymerase (Evrogen, Russia), 0.5 μL of 50× dNTP, 5 μL of a 10× Encyclo buffer, and 14.5 μL of mQ. 

PCR products were purified with the Cleanup Mini BC023S Kit (Evrogen, Russia). Sanger sequencing was carried out at Evrogen JSC (Moscow, Russia); the sequencing primers were the same as the amplification primers.

### 4.2. Sequence Alignment, Phylogenetic Analyses, and Molecular Dating

Sequences were aligned with MAFFT v.7 at default parameters [35], and the alignment was adjusted manually in PhyDe v.0.9971 [36]. Gaps were treated as missing data during the phylogenetic inference.

Two separate analyses were performed on nuclear and plastid DNA datasets via Bayesian inference (BI) and ML. According to the Akaike information criterion (AIC), the best-fitting model was the GTR + G model for the plastid dataset and the GTR + G + I model for the nuclear dataset, respectively. For the ML analyses, we employed RAxML v.8 [37]. Bootstrap analyses were conducted with 2500 replicates for ML. Due to the lack of statistically significant incongruence between nuclear and plastid trees (Figures S1 and S2), a combined sequence matrix was compiled for further analysis.

Divergence times for *Axyris* taxa were estimated using a Bayesian uncorrelated log-normal relaxed clock under a birth–death speciation process [38] for the combined dataset. We selected a normal distribution for the secondary calibration with a mean of 59.2 and standard deviation of 4.3, equivalent to the 95% HPD estimate from ref. [39] for the crown of Chenopodiaceae s.str. Bayesian analyses were conducted in BEAST v.2.6.7 [40]. Four Markov Chain Monte Carlo analyses with four chains were run for 20 million generations for every dataset, with sampling every 20,000 generations. Output log files were analyzed by means of TRACER v.1.6 [41] to assess convergence and ESS of all parameters; 15% of samples were removed as burn-in prior to combining the independent runs with the help of LOGCOMBINER v.2.6.7 [40]. The maximum clade credibility tree was generated using TREEANNOTATOR v.2.4.5 [40].

### 4.3. Biogeographical Analysis

Geographic distributions of all the studied species were inferred from herbarium specimens. Eight large geographic regions reflecting the worldwide distribution of Chenopodiaceae s. str. were coded as follows: A = Southern Siberia (Altai and Sayan Mountains and adjacent areas; Asiatic Russia), Mongolia, NW China (Xinjiang); B = Tian Shan and Pamir Mountains (Kyrgyzstan, East Kazakhstan, East Uzbekistan, Tajikistan); C = Greater Caucasus (Russia, Georgia, Azerbaijan); D = Himalayas/Tibet (Xizang, Qinghai and Sichuan provinces of China, Nepal, Bhutan, Jammu and Kashmir, Uttarakhand and Himachal Pradesh States of India); E = Eastern Siberia (Sakha Rep.); F = North America.

The BI gene trees were pruned to remove all duplicate accessions using the drop.tip function in the ape package [42]. Ancestral range estimation was conducted by means of the time-calibrated tree representing six species of *Axyris* with only one accession per species using “BioGeoBEARS” [43,44] in R v.4.1.3 [45]. The coded geographic data are displayed in Table 3.

We ran the analysis in accordance with a dispersal–extinction–cladogenesis model (DEC model), dispersal–vicariance model (DIVALIKE model), or BAYAREA model (BAYAREALIKE model) and examined a second run adding parameter “j” (founder-event speciation) for each biogeographic model. Out of the six models explored in this study, the DEC + J model was the best fit judging by the AIC and likelihood ratio test results (Table 4). The analyses were unconstrained (without possible dispersal routes or ancestral areas assumed a priori).

We allowed the inferred ancestor to occupy a maximum of three areas corresponding to the largest number of areas occupied by any extant species.

## Figures and Tables

**Table 1:** Voucher information and GenBank accession numbers for the species of *Axyris* and outgroups included in the phylogenetic analysis.

Species	Voucher	ITS	*rbcL*	*atpB-rbcL*	*trnL-F*
*Axyris amaranthoides*_1	Russia, Tyva Rep., Piy-Khemsky distr., nr Turan town, 11 August 2002, V.V. Nikitin et al. 1011 (LE)	ON775477	ON783748	ON783763	ON783778
*Axyris amaranthoides*_2	Russia, Amur prov., Blagoveschensk town, 50.270989, 127.565183, 7 September 2020, A. Sukhorukov 52 (MW)	ON775478	ON783749	ON783764	ON783779
*Axyris caucasica*_3	Russia, Karachaevo-Cherkessiya, Karachaevsky distr., nr Khurzuk vill., 11 August 2015, A.S. Zernov 7937 (MW0678817)	ON775479	ON783750	ON783765	ON783780
*Axyris caucasica*_5	Russia, Kabardino-Balkar Rep., Malka River, 13 July 2010, Bondarenko (LE)	ON775480	ON783751	ON783766	ON783781
*Axyris hybrida*_6	China, Xinjiang, [without date and collector], (XJBI00071453)	ON775481	ON783752	ON783767	ON783782
*Axyris hybrida*_7	Russia, Khakassiya Rep., Altaisky distr., Izykhslie Kopi vill., 21 August 2011, I. Shantser and N. Stepanova 111 (MHA)	ON775482	ON783753	ON783768	ON783783
*Axyris hybrida*_8	Russia, Buryat Rep., Baikal Nature Reserve, 21 August 2017, N.S. Gamova 2590 (MW0163600)	ON775483	ON783754	ON783769	ON783784
*Axyris hybrida*_9	Russia, Altai Rep., Chemalsky distr., nr Elanda vill., 13 August 1985, I. Pshenichnaya and G. Liventsova s.n. (MW0058817)	ON775484	ON783755	ON783770	ON783785
*Axyris hybrida*_10	Russia, Irkutsk prov., Olkhovsky distr., 31 July 2010, S.G. Kazanovsky 21796 (MW0160613)	ON775485	ON783756	ON783771	ON783786
*Axyris mira*_11	Nepal, Mugu Karnali valley, 16 August 1952, O. Polunin et al. 5251 (LE);	ON775486	ON783757	ON783772	ON783787
*Axyris prostrata*_12	Russia, Altai Rep., Kosh-Agach, Kurayskaya steppe, 24 August 2003, M. Knyazev s.n. (LE)	ON775487	ON783758	ON783773	ON783788
*Axyris prostrata*_13	Russia, Altai Rep., Kosh-Agach, 12 July 1990, R. Kamelin et al. s.n. (LE)	ON775488	ON783759	ON783774	ON783789
*Axyris prostrata*_14	Russia, Tyva Rep., Bay-Tayginsky distr., nr Kara-khol Lake, 1460 m, 23 July 1976, I. Krasnoborov et al. 498 (LE)	ON775489	ON783760	ON783775	ON783790
*Axyris sphaerosperma*_15	Russia, Altai Rep., Kosh-Agach, 1988, Kurbatsky et al. s.n. (LE)	ON775490	ON783761	ON783776	ON783791
*Axyris sphaerosperma*_16	Russia, Tyva Rep., Mongun-Tayginsky distr., 30 July 1981, M. Lomonosova and T. Akimenko 2882 (MW0058743)	ON775491	ON783762	ON783777	ON783792
**Outgroup**					
*Atriplex* *phyllostegia*	U. S. A., Nevada, Churchill Co., Zacharias 992 (UC)	HM005870	HM587590	HM587651	-
*Blitum antarcticum*	Chile, Tierra del Fuego, December 1971, Moore and Goodall s.n. (LE)	MH155315	MH632743	MH152573	MH632745
*Ceratocarpus arenarius*	No voucher	OP550131	OP554752	OP554754	OP554756
*Hablitzia tamnoides*	Bot. Gard. Bonn 3609-90 (BONN), Th. Borschz 3546	AY858590.1	AY270092.1	-	AY858600.1
*Krascheninnikovia lanata*	No voucher	OP550132	OP554753	OP554755	OP554757
*Neomonolepis spathulata*	USA, California, Susanville, August 1983, I.Yu. Koropachinsky et al. 404 (MHA)	MH675518	MH731232	MH152575	MH731230

**Table 2:** Primers and cycler programs used for DNA amplification.

Marker	Primer Sequences and Combinations	References	Cycler Program
ITS	ITS5 5′-GGA AGT AAA AGT CGT AAC AAG G-3′	[30]	95 °C for 5 min, 33 cycles of amplification (95 °C for 15 s, 55 °C for 30 s, 72 °C for 40 s), 72 °C for 5 min
	ITS4 5′-TCC TCC GCT TAT TGA TAT GC-3′		
*rbcL* (partial)	rbcLaF 5′- ATG TCA CCA CAA ACA GAG ACT AAA GC-3′	[31]	95 °C for 5 min, 35 cycles of amplification (95 °C for 10 s, 55 °C for 30 s, 72 °C for 40 s), 72 °C for 5 min
	rbcLaR 5′-GTA AAA TCA AGT CCA CCR CG-3′	[32]	
*atpB-rbcL* spacer	atpB-rbcL F 5′-GAA GTA GTA GGA TTG ATT CTC-3′	[33]	95 °C for 5 min, 35 cycles of amplification (95 °C for 20 s, 56 °C for 30 s, 72 °C for 60 s), 95 °C for 20 s, 56 °C for 80 s, 72 °C for 8 min
	atpB-rbcL R 5′-CAA CAC TTG CTT TAG TCT CTG-3′		
*trnL-F* spacer	Tab C 5′-CGA AAT CGG TAG ACG CTA CG-3′	[34]	95 °C for 5 min, 35 cycles of amplification (95 °C for 1 min, 50–65 °C [increasing in 0.3 °C per cycle] for 1 min, 72 °C for 4 min), 72 °C for 5 min
	Tab D 5′-GGG GAT AGA GGG ACT TGA AC-3′		
	Tab E 5′- GGT TCA AGT CCC TCT ATC CCC-3′		
	Tab F 5′ATI′ TGA ACT GGT GAC ACG AG 3′		

**Table 3:** The coding of the geographic areas of *Axyris* species and outgroups.

Taxon	Geographical Areas
*Axyris amaranthoides*	A
*Axyris caucasica*	C
*Axyris hybrida*	A, B
*Ax* *y* *ris mira*	D
*Axyris prostrata*	A, B, D
*Axyris sphaerosperma*	A, B, E
**Outgroups**	
*Atriplex phyllostegia*	F
*Blitum antarcticum*	F
*Ceratocarpus arenarius*	A
*Hablitzia tamnoides*	C
*Krascheninnikovia lanata*	F
*Neomonolepis spathulata*	F

**Table 4:** Results of the biogeographic analysis using BioGeoBEARS.

	LnL	Numparams	d	e	j	AIC	AIC_wt
DEC	−45.76	2	0.010	0.010	0	95.51	0.055
DEC + J	−41.92	3	0.0038	1.0 × 10^−12^	0.15	89.84	0.94
DIVALIKE	−50.19	2	0.010	0.010	0	104.4	0.0007
DIVALIKE + J	−50.14	3	0.010	0.010	0.0001	106.3	0.0003
BAYAREALIKE	−57.6	2	0.75	0.49	0	119.2	4.0 × 10^−7^
BAYAREALIKE + J	−54.08	3	0.42	0.30	0.55	114.2	4.9 × 10^−6^

## Data Availability

All the newly obtained DNA sequences were deposited in GenBank https://www.ncbi.nlm.nih.gov/genbank/ (accessed on 2 June 2022).

## Supplementary Materials

The following supporting information can be downloaded at https://www.mdpi.com/article/10.3390/plants11212873/s1, Figure S1: The ML phylogenetic cladogram of *Axyris* derived from nrITS; Figure S2: The ML phylogenetic cladogram of *Axyris* derived from the combined plastid matrix (*rbcL*, *atpB-rbcL*, and *trnL-F*).

## Appendix A

Siberian specimens of *Axyris* examined in different herbaria and used for the preparation of the maps:

*A. amaranthoides* map (the Siberian fragment only).

**Altaisky Krai** (selected): nr Barnaul, [without date], F.E. Zass s.n. (LE, TK); [Altaisky distr.] Komar, 29 July 1901, V.I. Vereschagin 541 (LE); Biysk town, 1920, A. Khrebtov 1393 (WIR); Aleysk town, 7 August 1925, I. Vykhodtsev s.n. (TK); [Kosikhinsky distr.] Ovchinnikovo vill., 27 August 1928, N. Koshurnikova s.n. (TK); [Suetsky distr.] nr Verkh-Suetka vill., 27 August 1932, V. Burdakova s.n. (TK); nr Rubtsovsk town, 18 August 1950, E. Vandakurova s.n. (NS); Ust’-Koksinsky distr., Katunsky Range, 27 August 1986, I. Artyomov and E. Bezvikonnykh 2 (NS); Blagoveschensky distr., Shimolino vill., 31 August 1988, M. Lomonosova and O. Zhdanova 10 (MW0058823); Blagoveschensky distr., Shimolino vill., 31 August 1988, M. Lomonosova and O. Zhdanova s.n. (NS); Pospelikhinsky distr., nr Krasnoyarskoe vill., 30 September 1999, I. Krasnoborov and D. Shaulo 85 (NS); Troitsky distr., nr Gornovoe vill., 8 September 2000, I. Krasnoborov et al. s.n. (NS); Klyuchevskoy distr., nr Klyuchi vill., 4 Oct 1999, I. Krasnoborov 130 (NS); Tselinny distr., nr Pobeda vill., 9 September 2000, I. Krasnoborov et al. 226 (NS); Krasnoschekovsky distr., Tigirek vill., 17 August 2001, V. Nikitin and I. Illarionova 179 (LE, MW).

**Altay Rep**.: [Maiminsky distr.] Ust’-Muny vill., 10 August 1927, N. Koshurnikova s.n. (TK); Shebalinsky distr., nr Chemal vill., 3 August 1972, V. Vašak s.n. (MHA); Shebalinsky distr., 21 August 1974, Yu.P. Surov and N.A. Sakharova s.n. (MW0058825); Ust’-Koksinsky distr., nr Yustik vill., 28 July 1984, D. Shaulo and A. Krasnikov 40 (NS); Shebalinsky distr., nr Elanda vill., 13 August 1985, I. Pshenichnaya and G. Liventsova s.n. (NS); Maiminsky distr., Chemal vill., 12 September 1990, M. Lomonosova et al. 193 (NS).

**Buryatiya Rep.** (selected): [Kabansky distr.] Kabansk, August 1912, Z. Belykh s.n. (LE); Eravninsky distr., Eravninskie Lakes, 6 August 1912, M. Korotkiy et al. 505 (LE); [Severobaikalsky distr.] Verkhneangarsk [Kumora], 9 August 1912, G. Poplavskaya 1274 (LE); [Muysky distr.] Muya River, 29 July 1914, M. Korotkiy and Z. Lebedeva 750 (LE); [Kabansky distr.] Posolskaya Station, 1 July 1915, Yu. Tsinserling 1486 (LE); Tunkinsky distr., Turan, 25 August 1929, M. Nazarov 12902 (LE, MW0058800); nr Verkhneudinsk [Ulan-Ude] town, 1930, B. Stazry s.n. (TK); [Djidinsky distr.] nr Naryn vill., 18 August 1934, P. Kursky s.n. (LE); [Barguzinsky distr.] nr Barguzin vill., 30 July 1942, L. Tyulina 146 (NSK); [Kabansky distr.] Mishikha vill., 18 August 1952, Andreeva 4820 (NSK); [Selenginsky distr.] nr Selenduma vill., September 1964, M.I. Maksimova s.n. (LE); [Ust’-Kyakhtinsky dist.] Ust’-Kyakhta vill., 26 July 1965, Peshkova and Skudenkova 2604 (NSK, TK); [Bichursky distr.] between Bichura and Okino Klyuchi vill., 16 August 1966, I. Buks s.n. (MWG); [Severo-Baikalsky distr.] nr Delakory vill., 25 August 1976, M. Ivanova and V. Keldin 356 (NSK); Pribaikalsky distr., nr Nesterovo vill., 15 August 1988, I. Krasnoborov and E. Pototskaya 108 (NS); Mukhorshibirsky distr., nr Podlopatki vill., 5 August 2013, L. Abramova et al. s.n. (MW0058807); Dzhidinsky distr., Baikal Nature reserve, 16 August 2017, N.S. Gamova 2537 (MW0163602); Kabansky distr., Baikal Nature Reserve, 18 August 2018, N.S. Gamova 2891 (MW0165073).

**Irkutsk prov**. (selected): Irkutsk, 1828, Turczaninov s.n. (LE); Nilova Pustyn’, 12 August 1873, Chersky et al. 342 (LE); Balagansky distr. [Cheremkhovsky distr.], Bazhey, 30 July 1902, N.I. Maltsev 285 (LE, NSK); [Zhigalovsky distr.] Znamenka, 26 July 1908, A. Krishtofovich s.n. (LE); [Osinsky distr.] Ust’-Osinskaya [Ust’-Altan], 1909, N. Maltsev 7308 (LE, MHA, MW0058805); [Osinsky distr.] Ust’-Osinskaya, 8 September 1909, N. Maltsev 1229 (LE); [Zhigalovsky distr.] Tutura, 14 August 1910, P. Aleksandrov 61 (LE); Kirensky distr., 15 July 1911, S.E. Kucherovskaya 624 (LE); [Usolsky distr.] Biliktuy, 1911, A. Petrov s.n. (LE); [Kachugsky distr.] Kachug, May 1914, M. Lukashina s.n. (LE); Baikal, Kultuk, 13 August 1915, Yu. Tsinzerling 3343 (LE); Baikal, nr Irkutsk, 27 July 1915, I.V. Larin 302 (LE); nr Tulun town, 30 July 1937, L. Kolokolnikov s.n. (NSK, TK); Kachugsky distr., nr Kharbatovo vill., 1948, N. Demikova and Z. Bespomestnykh s.n. (TK); [Chunsky distr.] Toreya station, 12 August 1952, A. Geld 47141 (NSK); [Osinsky distr.] Russkie Yanguty vill., 8 September 1955, A. Gorshkova 4888 (NSK); Cheremkhovsky distr., Inga vill., 1959, U. Krivotulenko 47120 (NSK); Nizhneilimsky distr., nr Kuklino vill., 13 August 1960, Lebedinov 357 (NSK); nr Angarsk town, 29 August 1971, I. Ivanov s.n. (WIR); Cheremkhovsky distr., 31 August 1971, I. Ivanov s.n. (WIR); nr Taishet town, 5 September 1979, I. Ivanov s.n. (WIR); [Kirensky distr.] Bubnovka vill., 10 July 1976, Zolottseva et al. 1033 (NSK); [Kazachinslo-Lensky distr.] Konets Lug vill., 29 July 1976, Zolottseva et al. 1276 (NSK); Katangsky distr., Nepa vill., 15 August 1976, Zolottseva and Vodopyanova 1770 (NSK); Irkutsk town, 30 August 1976, Z. Malysheva 362 (NSK); Kazachinsko-Lensky distr., Klyuchi vill., 7 September 1976, V. Siplivinsky 99 (LE); nr Irkutsk, September 1977, A.K. Skvortsov s.n. (MHA); Ziminsky distr., Maslyanogorsk vill., 4 July 1978, Kiselyova and Sergienko 335 (NSK); [Mamsko-Chuysky distr.] Slyudyanka vill., 12 August 1977, M. Ivanova 1583 (NSK); Cheremkhovsky distr., Tal’niki vill., 7 August 1978, Kiselyova and Kalashnikova 1046 (NSK); Nizhneudinsky distr., Porog vill., 2 August 1979, A. Kiselyova 1139 (NSK); mouth of Belaya River, 23 July 1983, Bondareva s.n. (NSK).

**Kemerovo prov.** (selected): Kuznetsky distr. [nr Novokuznetsk], 30 Jun 1912, N.I. Kuznetsov 1115 (LE); Mariinsky distr., 3 August 1912, S.E. Kucherovskaya 411 (LE); Topkinsky distr., nr Shishino vill., 12 July 1946, T. Grekhova s.n. (NS); [Tyazhinsky distr.] nr Pokrovka vill., 3 September 1946, T. Grekhova s.n. (NS); Leninsk-Kuznetsky distr., nr Arinichevo vill., 17 August 1956, L. Fedulina s.n. (TK); Belovsky distr., nr Urskoe vill., 9 August 1987, I. Makhatkov s.n. (NSK); Promyshlennovsky distr., nr Ust’-Kamenskaya vill., 4 September 1987, M. Lomonosova et al. s.n. (NS); Promyshlennovsky distr., nr Vaganovo vill., 6 September 1987, M. Lomonosova et al. s.n. (NS); Shatrovsky distr., nr Mekhonskoe vill., 6 July 1988, N. Naumenko s.n. (NS); Novokuznetsk town, 23 September 2021, M. Dzhus 2140 (MSKU).

**Khakassiya Rep**.: Abakan distr., August 1909, V. Titov s.n. (LE); [Beysky distr.] 5 September 1949, Cherepnin s.n. (LE); Bogradsky distr., nr Troitskoe vill., 4 August 1963, A. Korolyova and N. Minaeva s.n. (NS); Shirinsky distr., nr Borets vill., 19 July 1966, I. Neifeld and G. Kononona s.n. (NS); Ust’-Abakansky distr., Kamyzyak vill., 7 July 1967, G. Zvereva and I. Neyfeld s.n. (LE); Beysky distr., nr Kirba vill., 25 July 1967, I. Krasnoborov and V. Palchikov s.n. (LE, NS); Ust’-Abakansky distr., nr Kalinino vill., 16 Jun 1968, I. Neifeld and G. Chesnokova s.n. (NS); Tashtypsky distr., nr Tashtyp, 26 August 1968, I. Krasnoborov and E. Ershova s.n. (LE, NS); Bogradsky distr., nr Ust’-Erba vill., 3 August 1970, G. Girshovich and T. Kazakova s.n. (NS); Altaisky distr., nr Lukyanovka vill., 29 July 1974, I. Neifeld and E. Korovina 8465 (NS); Ordzhonikidzevsky distr., nr Kogunek vill., 24 July 1972, A. Koroleva and N. Sizova s.n. (NSK); Kezhemsky distr., nr Bolturino vill., 16 Jun 1978, D. Shaulo s.n. (NS); Kurtushibinsky Range, nr Usinskoe vill., 12 August 1980, D. Shaulo and T. Dongak 4326 (NS); Abakan town, 12 September 1989, M. Lomonosova et al. 110 (NS); Ust’-Abakansky distr., Krasnyi Kamen’ vill., 12 September 1989, M. Lomonosova et al. 111 (NS); Bogradsky distr., Borets vill., 13 September 1989, M. Lomonosova et al. 116 (NS); Tashtypsky distr., nr Bolshaya Seya vill., 20 August 1990, E. Ankipovich 18 (NS); Askizsky distr., Balankul Lake, 6 September 1991, E. Ankipovich and E. Lebedev 1 (NS).

**Krasnoyarsky Krai** (selected): Eniseysk town, 13 Oct 1876, M. Brenner s.n. (LE); Eniseysk town, 24 July 1891, A. Kytmanov s.n. (TK); [Novosyolovsky distr.] nr Anash vill., 3 August 1895, P. Chistyakov s.n. (TK); Kansk, August 1902, A. Shlyakhtin s.n. (LE); [Novosyolovsky distr.] nr Novosyolovo vill., 1903, V. Sapozhnikov s.n. (TK); Bogotol, 1903, A. Barsukov s.n. (LE); nr Krasnoyarsk, 7 September 1909, A.P. Yermolayev s.n. (LE); Nazarovsky distr., Altat vill., 18 July 1912, S. Kucherovskaya and V. Nekrasova 230 (LE); Achinsky distr., 24 July 1912, N.I. Kuznetsov 744 (LE); Minusinsky distr., 1913, A.V. Avdeeva s.n. (LE); nr Krasnoyarsk town, 1918, E. Konovalova s.n. (TK); [Kazachinsky distr.] nr Rozhdestvenskoe vill., 21 August 1921, anonymous s.n. (TK); [Ermakovsky dist.] nr Nizhneusinskoe vill., 10 August 1937, L. Londarenko et al. s.n. (TK); [Abansky distr.] nr Ust’-Yansk vill., July 1931, I. Arkhipov and V. Vandysheva s.n. (TK); Bogotolsky distr., nr Korobeinikovo vill., 5 September 1932, Z. Kaidarina et al. s.n. (TK); nr Bogotol town, 1934, V. Feldman s.n. (TK); Nazarovsky distr., nr Dorokhovo vill., August 1934, Grakhov s.n. (TK); Birilyussky distr., nr Arefyevo vill., 25 August 1934, V. Golubintseva s.n. (TK); Kuraginsky distr., nr Beryozovskoe vill., August 1936, M. Albitskaya and L. Londarenko s.n. (TK); Rybinsky distr., nr Churinovo vill., 5 August 1939, L. Kolokolnikov s.n. (TK); Dudinka, 15 August 1959, E.V. Dorogostayskaya s.n. (LE); Norilsk, 29 August 1959, E.V. Dorogostayskaya s.n. (LE); Bolshemurtinsky distr., nr Lakino vill., 28 July 1960, P. Filchukova and L. Sharapa s.n. (NS); Kuraginsky distr., nr Kochergino vill., 23 August 1962, A. Kuminova and G. Zvereva s.n. (NS); Abansky distr., nr Ustyansk vill., 20 August 1963, T. Vagina and N. Salnikova s.n. (NS); Rybinsky distr., nr Anzhinskoe vill., 25 August 1963, I. Krasnoborov and N. Alekseeva s.n. (NS); Karatuzsky distr., Mayka vill., 25 August 1964, I. Krasnoborov and V. Chayka s.n. (LE, NS); [Ermakovsky distr.] nr Idzhim vill., 9 August 1969, V. Nikitin et al. s.n. (WIR); Balakhtinsky distr., nr Elovka vill., 11 July 1973, I. Neifeld and N. Guskova s.n. (NSK); nr Kansk town, 3 August 1975, V. Amelchenko and N. Seleznyova s.n. (TK); Nazarovsky distr., nr Krasnye Sopki vill., July 1969, V. Nikitin et al. s.n. (WIR); [Evenkiysky distr.] 57 km W of Taimba vill., 20 July 1980, N. Bolshakov 530 (NSK); Ermakovsky distr., Aradansky Range, 12 September 1989, M. Lomonosova et al. 104 (NS).

**Kurgan prov**.: [Kurtamyshsky distr.] nr Kaminskoe vill., 31 July 1912, P. Krylov s.n. (TK); Lopatinsky distr., nr Lopatki vill., 19. July 1928, N. Ivanova and T. Tonishina 1093 (LE); Kurtamysh, 4 September 1928, N. Ivanova 1045 (LE); [Kataisky distr.] nr Ushakovskoe vill., 3 July 1980, K. Fedotova s.n. (NS); Zverinogolovsky distr., Zverinogolovskoe vill., 18 August 1989, M. Lomonosova and O. Zhdanova 129 (NS); Kurtamyshsky distr., nr Kurtamysh vill., 20 August 1989, M. Lomonosova and O. Zhdanova 22 (NS); [Zverinogolovsky distr.] nr Verkhnyaya Alabuga vill., 3 September 1990, N. Naumenko 3049 (NS).

**Novosibirsk prov.** (selected): Chany Lake, [without date] anonymous s.n. (LE); Kainsky distr. [Kuybyshevsky distr.], 10 August 1890, S. Korzhinsky s.n. (LE); Bolotninsky distr., between Bolotnoe town and Taskaevo vill., 30 August 1930, L. Pudovikova and V. Burdakova s.n. (TK); Maslyaninsky distr., nr Maslyanino vill., 14 September 1931, A. Zharkov s.n. (TK); Tatarsky distr., nr Kochnevka vill., 31 July 1955, V. Zhuravlyova and L. Malysheva s.n. (TK); [Ust’-Tarksky distr.] nr Ust’-Tarka vill., 2 September 1955, Peshkova s.n. (MHA, MOSP); Novosibirsk town, September 1956, L. Guryeva s.n. (NS); Novosibirsky distr., nr Payvino vill., 31 July 1963, N. Logutenko and A. Iksanova s.n. (NS); [Krasnoozyorsky distr.] nr Mokhnatyi Log vill., 20 August 1970, V. Nikitin et al. s.n. (WIR); Toguchinsky distr., 7 August 1973, A. Ronginskaya and O. Kazantsova 11 (LE); Maslyaninsky distr., Beryozovo vill., 18 July 1981, G. Dymina s.n. (NS); Zdvinsky distr., nr Chulym vill., 12 September 1985, L. Mironova and L. Dyukova s.n. (MHA); Zdvinsky distr., Chulym River, 15 September 1985, L. Mironova and E. Ershova s.n. (NS); Kargatsky distr., nr Mikhailovka vill., 16 September 1985, L. Mironova and D. Shaulo s.n. (NS); Toguchinsky distr., nr Mirnyi vill., 8 August 1986, I. Makhatkov s.n. (NSK); Ordynsky distr., nr Pichugi vill., 8 September 1986, M. Lomonosova et al. s.n. (MW0058833, NS); Toguchinsky distr., nr Stepnogutovo vill., 7 September 1987, M. Lomonosova et al. s.n. (NS); Kochenevsky distr., nr Kochenevo vill., 21 September 1987, M. Lomonosova and A. Krasnikov s.n. (NS); Iskitimsky distr., nr Ulybino vill., 15 September 1988, A. Krasnikov 544 (NS); Cherepanovsky distr., nr Ukrainka vill., 16 August 1994, O. Snytko and D. Shaulo 5 (NS); Kupinsky distr., Pokrovka vill., 6 September 1996, I. Krasnoborov and O. Zhirova s.n. (NS); Kupinsky distr., Nikitinka vill., 30 August 1997, I. Krasnoborov and D. Shaulo 70 (NS); Severny distr., Biaza vill., 24 August 2000, I. Krasnoborov and I. Artyomov 33 (NS); Kyshtovsky distr., nr Vyatka vill., 26 August 2000, I. Krasnoborov and I. Artyomov 79 (NS); Ust’-Tarksky distr., nr Ust’-Tarka vill., 29 August 2000, I. Krasnoborov et al. 119 (NS); Suzunsky distr., Inya River, 17 September 2002, D. Shaulo and I. Artyomov 80 (NS).

**Omsk prov.** (selected): Omsk, 10 August 1886, K. Golde s.n. (LE); Tyukalinsky distr., 4 August 1912, M. Ptaszycki 902 (LE); Cherlaksky distr., Tatarsky sovkhoz, 25 August 1971, T. Zhilina s.n. (WIR); [Poltavsky distr.] Krasnogorka vill., 23 August 1989, M. Lomonosova and O. Zhdanova 45 (NS).

**Sakha-Yakutiya Rep.** (selected): Amga river, Khayargas vill., 24 July 1902, P. Olenin 483 and 642 (LE); [Oymyakonsky distr.] Nelkan, 13 August 1903, I.M. Schegolev 1144 (LE); [Khangalassky distr.] Pokrovsk, 2 August 1905, Divnogorskaya s.n. (LE); [Verkhoyansky distr.] Borulahsky Nasleg, July 1912, R.I. Abolin 745 (LE); [Ust-Maysky distr.] Ust’-Maya, 23 July 1912, V. Drobov 538 (LE); between Yakutsk town and Aldan river, 20 August 1925, V. Drobov and A. Tarabunin s.n. (LE); [Megino-Kangalassky distr.] nr Maia vill., 12 August 1935, E. Bakanach s.n. (TK); Olyokminsky distr., nr Abaga vill., 2 September 1935, A. Kuminova and K. Sobolevskaya s.n. (TK); Tattinsky distr., 18 August 1951, V. Kuvayev 88 (MW0058791); Lensky distr., Peskovaya vill., 22 August 1952, E. Rubtsova s.n. (LE); [Mirninsky distr.] Tuoy-Khaya, 7 September 1958, I. D. Kaldushevsky 150 (LE); Yakutsk to Viluy along Lena river, [without date] A. Bronzov 228 (MW0058793); Olyokminsky distr., Yunkyur’ vill., 22 August 1967, I. Ivanov s.n. (WIR); Tomponsky distr., Tyoply Klyuch, 10 August 1968, A. Khokhryakov and M. Mazurenko s.n. (MHA); Alekseevsky distr., nt Ust’-Tatta vill., 23 August 1968, I. Ivanov s.n. (WIR); Vilyuisky distr., Chernyshevsk, 18 September 1968, I. Ivanov s.n. (WIR); Megino-Kangalassky distr., nr Byuteydyakh vill., 24 Jun 1969, I. Ivanov s.n. (WIR); Srednelensky distr., nr Kandy vill., 26 July 1969, I. Ivanov s.n. (WIR); [Tattinsky distr.] Cherkyokh vill., 29 July 1969, S.F. Nakhabtseva s.n. (MHA); Churapchinsky distr., 2 August 1969, I. Ivanov s.n. (WIR); nr Yakutsk, Tabaga headland, 13 August 1969, A. Khokhryakov and M. Mazurenko s.n. (MHA); nr Pokrovsk town, 17 August 1969, I. Ivanov s.n. (WIR); 54 km SW of Yakutsk town, 26 August 1969, I. Ivanov s.n. (WIR); nr Nyurba town, 22 August 1978, I. Ivanov s.n. (WIR); Chгrapchinsky distr., 18 August 1978, I. Ivanov s.n. (WIR); Khangalassky distr., Uryunbas vill., 31 August 1978, I. Ivanov s.n. (WIR); Olyokminsky distr., nr Chapaevo vill., Lena River, 5 August 1979, G. Dyuryagina and N. Arslanova 557 (NSK); [Khangalassky distr.] Nemyugyuntsy vill., 10 August 1979, I. Ivanov s.n. (WIR); Lensky distr., nr Peleduy vill., 14 August 1979, Ivanova and Zharchinskaya 843 (NSK); Olyokminsky distr., Kyatchi vill., 16 August 1979, I. Ivanov s.n. (WIR); Olyokminsky distr., Molbo landmark, 23 August 1979, I. Ivanov s.n. (WIR); Olyokminsky distr., Olbut vill., 27 August 1979, I. Ivanov s.n. (WIR); nr Olyokminsk town, 29 August 1979, I. Ivanov s.n. (WIR); Khangalassky distr., nr Khachikatsy vill., 24 August 1982, Vlasova and Bolshakov 4513 (NSK); Kobyaisky distr., nr Segyan-Kyuyol’ vill., 23 August 1985, Z. Malysheva 1358 (NSK); Namsky distr., nr Khamagatta vill., 20 August 2012, M. Lomonosova and E. Nikolin 790a (NS).

**Tomsk prov**. (selected): Tomsk, 30 August 1885, P.N. Krylov s.n. (LE, TK); [Kozhevnikovsky distr.] nr Urtamskoe vill., 23 July 1886, P. Krylov s.n. (TK); Kolpashevsky distr., 271 August 1927, N. Shipchinsky 604 (LE); Asinovsky distr., nr Nizhnie Sokoly vill., 27 August 1949, A. Andrievskaya s.n. (TK); nr Kolpashevo town, 1 September 1957, V. Dobychin s.n. (TK); Tomsky distr., Yar vill., 2 August 1986, E.D. Lapshina s.n. (LE); Kozhevnikovsky distr., nr Urtam vill., 27 August 1999, V. Amelchenko s.n. (NSK).

**Tyumen prov**.: Tyumen, 1883, herb. Trautvetter 203 (LE); nr Tobolsk town, [without year] L. Lugovsky s.n. (TK); Yalutorovsky distr., 3 July 1912, V.I. Svitich 1359 (LE); nr Ishim, 21 July 1912, B.N. Gorodkov s.n. (LE); Tobolsk, 1915, S. Mamayev 451 (LE, NSK); [Vikulovsky distr.] Vikulovo, 30 July 1915, V.A. Varentsov s.n. (LE); Khanty-Mansi Okrug, Kondinsky distr., Tumany Lakes, 30 August 1946, F.V. Sokolov 301 (LE).

**Tyva Rep**. (selected): Ulug-Khemsky distr., Kamenny range, 17 August 1968, I. Bagrintseva s.n. (MHA); nr Turan town, 9 August 1969, V. Nikitin et al. s.n. (WIR); Kaa-Khemsky distr., nr Ilyinka vill., 18 July 1972, E. Penkovskaya and L. Kupalova 1478 (NS); nr Seserlig vill., 31 July 1974, L. Danilyuk et V. Kurlaev 2926 (LE, MHA, NS); Kyzylsky distr., nr Cherbi vill., 28 July 1975, V. Korotkova and D. Konovalov 1831 (LE, MHA, MW0058816, NS); Piy-Khemsky distr., nr Oyun-Shivi vill., 3 August 1975, N. Iskakova 2517 (LE, MW0058813); Todzhinsky distr., nr Toora-Khem vill., 27 July 1978, M. Lomonosova et al. 267 (LE); Todzha, nr Azas Lake, 23 July 1978, M. Lomonosova and V. Khanminchun 63 (LE, MHA, MW0058811, NS); Tandinsky distr., nr Balgazyn vill., 12 August 1978, I. Neyfeld and N. Larina s.n. (LE, NS); Piy-Khemsky distr., nr Turan town, 11 August 2002, V.V. Nikitin et al. 1011 (LE); Todjinsky distr., nr Kadysh-Khol Lake, 31 July 2004, D. Shaulo and N. Chinkhotyan 88 (NS).

**Zabaikalsky Krai** (selected): Nerchinsk, 1892, F. Karo 236 (MW0058802); [Mogochinsky distr.] Bushuley, 20 July 1908, I.V. Novopokrovsky s.n. (LE); [Chernyshevsky distr.] Milgidun, 26 July 1908, I. Novopokrovsky s.n. (LE); Vitim river, 30 July 1909, anonymous 119 (LE); [Sretensky distr.] Gorbitsa vill., 2 August 1909, N.I. Kuznetsov 1792 (LE, NS); nr Chita, 1911, Petrov s.n. (LE); [Karymsky distr.] Karymskoe, 13 August 1928, T.I. Solokhin s.n. (LE); [Chitinsky distr.] Sokhondo vill., 1930, K.F. Yakovlev s.n. (MW0058794); [Olovyanninsky distr.] Olovyannaya st., 1 August 1931, M. Nazarov 14121 (MW0058798); Shilkinsky distr., 24 August 1939, E. Pospelov s.n. (TK); Aginskoye, 17 August 1948, A.A. Fedorov et al. s.n. (LE); Byrkinsky distr., nr Byrka vill., 4 August 1949, N. Verkhovinskaya and N. Listova s.n. (TK); Zabaikalsky distr., Kharanor vill., 8 September 1950, P.A. Smirnov and Shilkina s.n. (MW0058799); [Mogochinsky distr.] Pokrovka vill., 24 August 1953, G. Peshkova and B. Khantagaev 5047 (NSK); Beklemishevsky [Chitinsky] distr., Tasey vill., 20 July 1967, N.I. Filina and V.R. Filin s.n. (MW0058804); Kodar Range, 16 August 1978, A. Chepurnov s.n. (NSK); Nerchinsky distr., Priiskovyi vill., 28 August 1987, S. Rumyantsev and I. Shantser s.n. (NSK); [Borzinsky distr.] Borzya town, 5 August 1988, V.D. Bochkin et al. s.n. (MHA).

*Axyris hybrida* (Russian fragment only)

**Altaisky Krai:** [Talmensky distr.] nr Ozyorki vill., 20 July 1891, P. Krylov s.n. (TK); nr Barnaul, 10 August 1908, E. Rodd 6 (LE); nr Zmeinogorsk town, 1911, A. Anokhina s.n. (TK); [Krasnoschekovsky distr.] Maralikha vill., 8 July 1913, N.I. Kuznetsov 1747 (LE); [Biysky distr.] between Verkhnekatunskoe and Srostki, 1915, P.N. Krylov s.n. (LE, NS); Smolensky distr., nr Smolenskoe vill., July 1930, A. Vinogradova and A. Filatova s.n. (TK); Soloneshensky distr., nr Elinovo vill., 9 September 1931, Z. Kaidarina and G. Ashikhmina s.n. (TK); Belokurikha town, July 1933, L. Bedro s.n. (TK); Mamontovsky distr., nr Mamontovo, 19 August 1946, E. Vandakurova and G. Kobzar s.n. (LE, NS); Troitsky distr., nr Troitskoe vill., 6 September 1980, I. Krasnoborov and V. Khanminchun 314 (NS); Troitsky distr., nr Gornovoe vill., 8 September 2000, I. Krasnoborov et al. 146 (NS).

**Altai Rep.** (selected): Chuya river, 1837, Politov 2217 (LE); [Shebalinsky distr.] Myyuta vill., 10 August 1904, B. Klements 577 (LE); [Ust’-Kansky distr.] Ust’-Muta, 26 July 1913, N.I. Kuznetsov 2846 (LE); Ust’-Koksa, 2 August 1924, P.F. Serdyukov 102 (LE); Katun’ river, 5 August 1925, V. Sheludyakova 8 (LE); [Ust’-Kansky distr.] nr Chyorny Anuy, 17 July 1929, E.G. Pobedimova 192 (LE); [Ulagansky distr.] nr Teletskoe Lake, nr Chiri vill., 25 September 1936, M. Khomutova 168 (MOSP); Kosh-Agachsky distr., Buraty, 8 August 1950, A. Kuminova and G. Pavlova s.n. (LE); [Ust’-Koksinsky distr.] ‘nr Ust’-Koksa vill., 8 August 1953, E. Pobedimova and S. Kolomoytseva s.n. (LE); [Ust-Koksinsky distr.] nr. Bashtala vill., 24 August 1953, E. Pobedimova and S. Kolomoytseva s.n. (LE); [Ongudaisky distr.] Onguday vill., 6 July 1931, E. Steinberg 1943 (LE); Ongudaysky distr., nr Khabarovka vill., 5 September 1969, I. Krasnoborov s.n. (NSK); Ulagansky distr., Aktash vill., 23 August 1977, N. Zolotukhin 82 (MW0058824); Ulagansky distr., Ust’-Ulagan, 1220 m, 10 August 1983, M. Danilov and L. Gunderina s.n. (LE); Ust’-Koksinsky distr., nr Katanda vill., 4 September 1983, Yu.M. Maskaev s.n. (MW0058770); Ongudaisky distr., Seminsky Range, 1230 m a.s.l., 20 July 1984, I. Pshenichnaya and G. Liventsova s.n. (NS); Ust’-Koksinsky distr., nr Ust’-Koksa vill., 14 August 1984, D. Shaulo 102 (MHA); Ust’-Kansky distr., nr Ust’-Kan vill., 17 August 1984, D. Shaulo and O. Zhdanova 105 (NS); Chemalsky distr., nr Elanda vill., 13 August 1985, I. Pshenichnaya and G. Liventsova s.n. (MW0058817, NS); Ongudaisky distr., Khabarovka vill., 11 September 1990, M. Lomonosova et al. 192 (NS); Ust’-Kalmansky distr., nr Novokalmanka vill., 13 August 1998, I. Krasnoborov and A. Shmakov 18 (NS).

**Buryatiya Rep.** (selected): [Barguzinsky distr.] nr Barguzin, 1934, herb. Fischer s.n. (LE); Verkhneudinsk [Ulan-Ude] town, [without date] Sedakov s.n. (LE); Tunkinsky distr., 8 August 1902, V. Komarov s.n. (LE); [Kyakhtinsky distr.] Dureny, 19 August 1905, T. Mikhno s.n. (LE); [Severo-Baikalsky distr.] nr Verkhneangarsk [Kumora] town, 17 July 1912, V. Sukachev and G. Poplavskaya 777 (LE, MW0058806); [Eravninsky distr.] Eravninskie Lakes, 30 July 1912, M. Korotkiy et al. 457 (LE); Khaim, 30 July 1913, G. Poplavskaya et al. 2103 (LE); [Pribaykalsky distr.] Tataurovo, 6 August 1913, G. Poplavskaya 2417 (LE); nr Verkhneudinsk [Ulan-Ude], 6 August 1913, G. Poplavskaya et al. 2451 (LE); [Kyakhtinsky distr.] Ust’-Kiran, 12 July 1915, T. Mikhno s.n. (LE); [Kabansky distr.] Selenginsk vill., 4 August 1915, G. Poplavskaya and V. Sukachev 3202 (LE); Barguzinsky distr., Bolshoy Ushkaniy Island, 9 August 1916, I. Larin and G. Kanevsky 772 and 773 (LE); Barguzinsky distr., Baikal, Svyatoy Nos Peninsula, 23 August 1916, I. Larin and G. Kanevsky 961 (LE); [Dzhidinsky distr.] Armak vill., 11 Oct 1932, M. Nazarov 15398 (MW0058767, MW0058795); [Djidinsky distr.] nr Naryn vill., 18 August 1934, P. Kursky s.n. (LE); Zaigraevsky distr., nr Kurbinskoe vill., 12 August 1948, L. Sergievskaya s.n. (TK); Pribaikalsky distr., nr Goryachinsk vill., 12 August 1948, S. Gudoshnikov s.n. (TK); Selenginsky distr., nr Zalan vill., 29 July 1952, Sultanova s.n. (NSK); [Ivolginsky distr.] Ivolga river, 7 August 1955, P. Sokolov 333 (LE); Duldurginsky distr., 31 July 1956, V. Kuvaev 1354 (TK); [Tunkinsky distr.] Turan vill., 21 August 1958, L. Malyshev 322 (LE, NSK); Tunkinsky distr., nr Mondy vill., 15 August 1963, Alyanskaya et al. s.n. (MHA, MOSP, LE, NS); [Selenginsky distr.] Zhargalanta vill., September 1963, M. Maksimova s.n. (LE, TK); [Bichursky distr.] between Bichura and Okino Klyuchi vill., 16 August 1966, I. Buks s.n. (MWG); [Kabansky distr.] mouth of Manturikha River, 16 August 1975, A. Kiselyova and N. Vlasova 1057 (NSK); [Kabansky distr.] nr Boyarsky vill., 11 August 1975, A. Kiselyova 906 (NSK); Severobaikalsky distr., 12 August 1976, Shibanova and Sivoklokova s.n. (MW0058759); [Selenginsky distr.] nr Gusinoe Ozero vill., 1982, G. Kupatadze s.n. (MOSP); [Djidinsky distr.] nr Verkhnee Beloe Lake, 10 September 1985, G. Peshkova 8554 (NSK); Eravninsky distr., nr Tuldun vill., 5 August 1988, I. Krasnoborov and M. Danilov 57 (NS); Barguzinsky distr., nr Monakhovo vill., 10 August 1988, I.M. Krasnoborov 81 (MHA, MW0058777, NS); Djidinsky distr., nr Nizhniy Torei vill., 9 July 2007, E. Korolyuk and A. Korolyuk s.n. (NS); Djidinsky distr., Samkhak, 1141 m, 2 August 2013, N. Gamova 0782 (MW0156350); Baikal Nature reserve, Temnik river, 21 August 2017, N. Gamova 2590 (MW0163600).

**Irkutsk prov**.: Irkutsk, 1828, Turczaninov s.n. (LE) together with *A. amaranthoides*; Nizhneudinsk, 25 July 1902, B.P. Bogorodsky 541 (MW0058764); [Balagansky distr.] Konovalovo, 7 August 1903, D. Litvinov 813 (LE); [Cheremkhovsky distr.] Bazhey vill., 29 July 1905, N. Maltsev s.n. (LE); [Osinsky distr.] Osa vill., 8 September 1909, N. Maltsev 1229 (LE, TK) and 7308 (NS, TK); [Zhigalovsky distr.] Tutura vill., 10 August 1910, P. Aleksandrov 288 (LE); [Kachugsky distr.] nr Kachug vill., 2 August 1915, G. Kanevsky 316 and 377 (LE, LECB); Baikal Lake, Kultuk vill., 13 August 1915, Yu. Tsinzerling 3343 (LE); Kachugsky distr., nr Kharbatovo vill., 1948, N. Demikova and Z. Bespomestnykh s.n. (TK); [Olkhonsky distr.] nr Khuzhir vill., 9 August 1960, L. Malyshev and A. Sokolnikov 407 (NSK); [Bokhansky distr.] nr Bokhan vill., 24 July 1966, Peshkova and Lysenko 1516 (NSK, TK); Olkhon Island, 16 August 1976, E. Musayev et al. s.n. (MW0105052); Irkutsky distr., Koty vill., 24 September 1976, V. Siplivinsky 97 (LE); Olkhonsky distr., Sarma vill., 17 August 1986, M. Ivanova 384 (MHA, NS); Irkutsky distr., Baikal Lake, NE from Listvyanka vill., 14 September 1989, M.V. Kostina and L.A. Kramarenko s.n. (MHA); Olkhovsky distr., Kosaya Step’ vill., 31 July 2010, S.G. Kazanovsky 21796 (MW0160613).

**Kemerovo prov**.: Guryevsk town, [without year, probably late 19th century], F. Zass s.n. (TK).

**Khakassiya Rep.** (selected): [Shirinsky distr.] Shira Lake, 29 July 1897, Yu. Vagner s.n. (LE); Oznachennoye [Sayanogorsk], 7 September 1931, M. Iljin and B. Ovchinnikov 429 (MW0058772, NSK); [Ust’-Abakansky distr.] Ust’-Byur vill., August 1934, Z. Tarchevskaya and S. Gluzdakov s.n. (TK); Bogradsky distr., nr Sonskoe vill., 27 August 1936, K. Sobolevskaya and L. Zaitseva s.n. (TK); Shirinsky distr., nr Shira vill., 1 August 1966, I. Neifeld and N. Alekseeva s.n. (NS); Altaisky distr., nr Smirnovka vill., 8 August 1967, N. Alekseeva and T. Lamanova s.n. (NS); Ust’-Abakansky distr., nr Charkov vill., 26 July 1969, I. Neifeld and V. Krotova 8463 (NS); Bogradsky distr., nr Bei-Buluk vill., 14 August 1971, G. Girshovich and L. Parshulina s.n. (NS); Altaisky distr., Abakan river, Izykhskie Kopi vill., 300 m, 21 August 2011, I. Shantser and N. Stepanova 111 (MHA).

**Krasnoyarsky krai**: nr Krasnoyarsk, [without date] anonymous s.n. (LE); Minusinsky distr., Bystraya vill., August 1874, N. Martyanov 497 (LE); nr Minusinsk, 20 August 1902, N. Martyanov exs. 7309 (LE, MHA, MW0058768, NS); Minusinsky distr., nr Bystraya vill., 6 August 1964, G. Zvereva and N. Drobyshevskaya s.n. (NS); Krasnoturansky distr., nr Krasnoturansk vill., 25 August 1965, A. Kuminova and N. Alekseeva s.n. (NS); [Ermakovsky distr.] nr Idzhim vill., 9 August 1969, V. Nikitin et al. s.n. (WIR) together with *A. amaranthoides*; Novosyolovky distr., nr Tolsty Mys vill., 6 August 1972, V. Krechetov and T. Kuznetsova s.n. (NS); Novosyolovsky distr., Sharypovsky distr., Dubinino, 13 August 1974, I. Krasnoborov et al. 491 (LE, NS); Kurtushibinsky Range, August 1980, D. Shaulo and T. Nalpina 3104 (LE, NS); Uzhursky distr., Uchum Lake, 13 September 1989, M. Lomonosova et al. 122 (NS); Sayanskoe Water Reservoire, 24 July 2009, A. Sonnikova s.n. (NS).

**Novosibirsk prov**.: [Suzunsky distr.] nr Shipunovo vill., 26 July 1930, A. Saltykova and Z. Kaydarina s.n. (NS); [Kochenyovsky distr.] nr Bun’kovo vill., 25 August 1934, A. Konusova s.n. (TK); Zdvinsky distr., nr Gorodische vill., 2 August 1946, T. Popov and N. Shabovich s.n. (NS); Toguchinsky distr., nr Yurty vill., 4 September 1987, M. Lomonosova et al. s.n. (NS).

**Tyva Rep**.: Ovyursky distr., Chazy river, 1 September 1946, A. Shreter 59 (MW0058822); Bai-Taiginsky distr., Kara-khol Lake, 27 August 1962, S. Gudoshnikov and F. Balabanov s.n. (TK); Kaa-Khemsky distr., nr Ilyinka vill., 14 July 1972, E. Penkovskaya and L. Eremenko 1724 (LE, MHA); Tsagan-Shibetu Range, 1500 m, 14 July 1972, I. Krasnoborov and T. Grushevskaya 540 (LE); Erzinsky distr., nr Erzin vill., 18 July 1972, S. Timokhina and L. Daniluyk s.n. (MHA); Tes-Khemsky distr., Tannu-Ola Range, 1750 m, 7 August 1972, V. Khanminchun et al. 100358 and 100364 (LE, NS, TK); Ovyursky distr., nr Ak-Chyraa vill., 10 August 1973, S. Timokhina and M. Sakovich 1413 (LE, MHA, MW0058776); [Tere-Kholsky distr.] nr Chirgalandy vill., 28 July 1974, V. Amelchenko and B. Sviridenko s.n. (TK); nr Seserlig vill., 30 July 1974, M. Lomonosova and L. Danilyuk 3157 (LE, MHA, MW0058773, NS); Bay-Tayginsky distr., nr Kara-hol Lake, 1650 m, 18 July 1976, I. Kransoborov et al. 497 (LE, NS); Ovyursky distr., nr Sagly vill., 24 July 1976, E. Korotkova and E. Rubtsova 1836 (NS); Tanzhu-Ola, nr Chadan town, 750 m, 31 July 1976, V. Grubov et al. 499 (LE); Ovyursky distr., nr Khandagaity vill., 6 August 1976, E. Korotkova and T. Polyakova 1833 (MHA); Ovyursky distr., nr Sagly vill., 5 August 1977, E. Korotkova and M. Danilov 476 (LE); Dzun-Khemchiksky distr., nr Bazhyn-Alaak vill., 17 August 1977, E. Korotkova and O. Emelyantseva 72 (LE, NS); Ovyursky distr., nr Khandagaity vill., 11 August 1977, E. Korotkova and M. Danilov 477 (NS); Kaa-Khemsky distr., 22 July 1978, D. Shaulo 1364 (LE, MW0058769); Tes-Khemsky distr., nr Bert-Dag vill., 4 August 1978, I. Neifeld s.n. (NS); Piy-Khemsky distr., nr Arzhan vill., 8 July 1979, M. Lomonosova and S. Molokova 373 (MW0058775); Mongun-Taiginsky distr., Chikhachyov Range, 2500 m a.s.l., 3 August 1982, I. Krasnoborov and L. Mironova s.n. (NS); Erzinsky distr., 20 km SW from Erzin vill., 24 August 1985, A.M. Laidyp s.n. (MHA); Kyzylsky distr., Khadyn Lake, 3 September 1989, M. Lomonosova et al. 53 (NS); Erzinsky distr., nr Bai-Khol Lake, 6 September 1989, M. Lomonosova and D. Shaulo 72 (NS).

**Zabaykalsky Krai** (selected): between Onon and Argun’ rivers, 1856, G. Radde 9 (LE); Nerchinsk, July 1893, Stukov 76 (LE); [Nerchinsky distr.] Byankino vill., 1 July 1901, A. Fadeev s.n. (LE); Buryatskaya station, 27 July 1903, D. Litvinov 532 (LE); Aginskoe, July 1908, G. Stukov 156 (LE); [Slyudyansky distr.] nr Kultuk vill., 3 August 1910, P. Orlov s.n. (TK); [Zabaikalsky distr.] Abagaytuy vill., 24 July 1911, V. Smirnov 5722 (LE); [Karymsky distr.] nr Mayaki vill., 5 August 1925, B. Zamoshnikov s.n. (TK); Zabaikalsky distr., Matsievskaya station, 1 September 1930, M.T. Ivanova 456 (LE); [Priargunsky distr.] nr Urulyungui vill., 12 July 1931, A. Vinogradova and E. Chebakova s.n. (TK); nr Chita town, 28 August 1938, L. Sergievskaya s.n. (TK); Borzinsky distr., nr Borzya vill., 9 August 1949, L. Sergievskaya et al. s.n. (TK); Ononsky distr., 12 September 1950, V. Kuvaev 281 (LE); Ulyotovsky distr., nr Shebartui vill., 21 July 1952, L. Sergievskaya and L. Obolentsev s.n. (TK); Olovyanninsky distr., nr Olovyannaya vill., 17 September 1956, V. Kuvaev 2881 (TK); Aginsky distr., 27 August 1958, R. Pimenova 258 (MW0058760); [Ononsky distr.] Stary Chindant vill., 8 August 1964, G. Peshkova and L. Ovchinnikova s.n. (NSK); Beklemishevsky [Chitinsky] distr., Tasey vill., 20 July 1967, N. Filina and V. Filin s.n. (MW0058766); Zun-Torei Lake, 1967, anonymous s.n. (TK); from Borzya to Balei town, 7 August 1971, I. Ivanov s.n. (WIR); Aginskoe vill., 12 August 1977, N. Alyanskaya and T. Sofeikova s.n. (MHA); Aginsky distr., Nozhiy Lake, 649 m a.s.l., 18 July 2008, A. Konovalov and M. Isaykina 23829 (NSK); Ononsky distr., 15 km W of Ust’-Imalka vill., 653 m a.s.l., 25 July 2008, M. Isaykina and N. Pazdnikova 24528 (NSK); Borzinsky distr., 17 km W of Sherlovaya Gora vill., 954 m a.s.l., 2 August 2008, V. Chepinoga et al. 25841 (NSK).

*Axyris prostrata* (Russian fragment only)

**Altaisky Krai:** nr Barnaul, [without date] F.E. Zass s.n. (LE, TK).

**Altai Rep**. (selected): Chuya, [without date], A. Bunge 1435 (LE); Kosh-Agach, 22 August 1931, G. Sumnevich s.n. (LE); Akalakha river, 29 July 1931, B. Shishkin et al. s.n. (LE); Ukok Plateau, Akkol River valley, 23 July 1955, A. Kuminova and N. Listova s.n. (NS); Ulagansky distr., nr Aktash vill., 28 July 1981, M. Danilov and N. Kolesnikova s.n. (MW0058755, NS); Kosh-Agach, Chikhachev range, 2300 m, 7 July 1982, A. Maneev and A. Krasnikov s.n. (MHA, MW0058758, NS); Kosh-Agach, 12 July 1990, R. Kamelin et al. s.n. (LE); Kosh-Agach, Kurayskaya steppe, 24 August 2003, M. Knyazev s.n. (LE); Kosh-Agachsky distr., Zhana-Aul vill., 3 August 2009, E. Korolyuk 67 (NS).

**Buryatiya Rep.:** Tunkinsky distr., Sentsy river, 4 August 1932, M. Nazarov 15095 (MW0058745); Tunkinsky distr., Mondy vill., 12 September 1936, V.I. Smirnov s.n. (LE); [Okinsky distr.] Orlik vill., 22 August 1960, L. Malyshev 406 (LE, NSK); Tunkinsky distr., Mondy vill., 29 August 1963, N. Kozhevnikova s.n. (MHA, NS).

**Irkutsk prov.:** Irkutsky distr., Baikal Lake, NW from Listvyanka vill., 14 September 1989, M.V. Kostina and L.A. Kramarenko s.n. (MHA).

**Krasnoyarsky Krai**: [Balakhtinsky distr.] Maryasovo vill., 19 August 1912, I.V. Kuznetsov 916 (LE); Kansky distr., nr Geogrievka vill., 26 July 1931, I. Arkhipov and V. Vandysheva s.n. (TK).

**Tyva Rep**. (selected): Ovyursky distr., nr Torgalyg vill., 16 August 1969, V. Grankina s.n. (NS); Erzinsky distr., nr Naryn vill., 1258 m, 13 July 1972, I. Krasnoborov and N. Bezyazykova 3625 (LE, NS); Erzinsky distr., Tere-khol Lake, 1150 m, 26 July 1972, S. Timokhina and M. Sakovich 2619 (LE, NS, NSK); nr Teeli vill., 9 July 1976, S. Timokhina and S. Kochergin 359 (LE, MW0058758); Bai-Taiginsky distr., nr Kara-khol Lake, 1460 m, 23 July 1976, I. Krasnoborov et al. 498 (LE, NS); Barun-Khemchiksky distr., nr Khondelen vill., 11 August 1976, I. Neifeld and N. Enishevskaya s.n. (NS); Tannu-Ola, nr Chadan town, 7 August 1978, I. Krasnoborov 87 (LE); Tes-Khemsky distr., nr Samagaltay vill., 16 July 1978, A. Kuminova and I. Neyfeld s.n. (LE); Erzinsky distr., Tere-khol Lake, 29 July 1979, M. Lomonosova and A. Krasnikov 962 (MHA); Mongun-Taiginsky distr., Chikhachyov range, 20 July 1980, A. Krasnikov et al. s.n. (MHA, NS); Mongun-Taiginsky distr., Mogen-Buren River valley 2100 m a.s.l., 1 August 1982, I. Krasnoborov and L. Mironova s.n. (NS); Ovyursky distr., nr Sagly vill., 26 July 1993, I. Krasnoborov 315 (NS).

*Axyris sphaerosperma* (general distribution)

**China** (first records): Irenchabirga, 9000 ft, 10 September 1879, A. Regel (LE); Zaisan expedition, [Xinjiang prov., Habahe county] Kobuk River basin, 20 July 1914, V. Sapozhnikov s.n. (LE).

**Kazakhstan**: Kungey-Alatoo Mts., Tau-Chilik [Tau-Shelek], 3150 m, 11 August 1944, V. Goloskokov s.n. (LE); East Kazakhstan, 60 km NW of Ayaguz town, Chingiztau Range, 18 July 1966, V. Vasilevich et al. 798 (LE).

**Kyrgyzstan**: Naryntau Range, nr Naryn River, 2800 m a.s.l., B. Ovchinnikov and M. Usov 321 (LE).

**Mongolia** (first record): Mongolian Altai, Bayan-Ulgii prov., Tavan-Bogd Mts., Tsagaan-Gol River basin, 4 August 2001, I. Krasnoborov, A. Shmakov, and D. German 82 (NS0009424).

**Russia: Altai Rep.** (selected): Chuya, 1837, Politov 348 (LE); Ulagansky distr., Bashkaus river basin, 24 July 1940, L. Obolentsev s.n. (TK); Kosh-Agachsky distr., Sayluygem steppe, 6 August 1950, A. Kuminova and G. Pavlova s.n. (MHA); [Kosh-Agachsky distr.] nr Ukok Plateau, 25 August 1985, V. Zuev 1922 (NSK); Kosh-Agach, July 1988, V.I. Kurbatsky et al. s.n. (LE); Ulagansky distr., Aktash vill., 6 September 1990, M. Lomonosova et al. 154 (NS). **Sakha-Yakutiya Rep**.: [Verkhoyansky distr.] Borulahsky Nasleg, 1 July 1912, R.I. Abolin 527 (LE); Yakutsky okrug, Eren-Kyol’ landmark, 14 July 1912, R.I. Abolin 679 (LE); Yakutsk okrug, Lena river valley, Boskon Island, 17 July 1912, G.I. Dolenko 680 (LE); [Gornyi distr.] Maltaninsky Nasleg, 8 August 1912, R.I. Abolin 873 (LE); [Khangalassky distr.] Beguyrskaya station, 20 August 1912, V. Drobov 618 (LE); [Khangalassky distr.] Beguyrskaya station, 23 August 1912, V. Sokolov 1666, 1700 and 1701 (LE); between Yakutsk and Aldan, 14 August 1925, V. Drobov and A. Tarabunin 1277 (LE); Yakutsk to Viluy along Lena river, 24 September 1932, A. Bronzov s.n. (MW0058740); [Yakutsk town] Markha, 15 August 1940, M. Karavaev s.n. (TK); Tattinsky distr., 20 August 1951, V. Kuvayev 90 (MW0058741, NS); [Yakutsk town] Kandagassy, Lena River, September 1959, M. Karavaev s.n. (NSK); [Oymyakonsky distr.] Indigirka river, Tomtor vill., 6 August 1960, M. Karavayev s.n. (MW0058742); Oymyakonsky disr., Tomtor vill., 28 August 1983, A. Khokhryakov s.n. (NS); Pokrovsky Trakt, [Yakutsk town] Vladimirovka vill., 23 August 2012, M. Lomonosova and E. Nikolin 825 (NS); Khangalassky distr., nr Ulakhan-An vill., 24 August 2012, M. Lomonosova and E. Nikolin 857 (NS). **Tyva Rep**.: Elegest river, Bai-Khaak, 26 July 1916, G. Miklashevskaya s.n. (LE); Tes-Khemsky distr., West Tannu-Ola Range, Kholu River basin, 7 August 1972, V. Khanminchun et al. 100353 (NS); Ovyursky distr., West Tannu-Ola Range, 29 July 1975, V. Khanminchun and D. Shaulo 1086 (NS); Bai-Taiginsky distr., nr Kara-hol Lake, 1650 m, 26 July 1976, I. Krasnoborov et al. 496 (LE, NS); Mongun-Tayginsky distr., 30 July 1981, M. Lomonosova and T. Akimenko 2882 (LE, MW0058743, NS).

**Tajikistan**: nr Peter 1st Range, 12 km SW of Kazak vill., Yashil-kul Lake, 3000 m a.s.l, 13 August 1963, T. Strizhova 3239 (LE).
