# Supplementary material for: Biogeography and Systematics of the Genus Axyris (Amaranthaceae s.l.)
Source: Plants (Basel). 2022 Oct 27;11(21):2873. doi: 10.3390/plants11212873 (PMC9657989; doi:10.3390/plants11212873)
Supplement: Supplementary file 1 [file plants-11-02873-s001.zip › plants-1984639-supplementary.pdf]

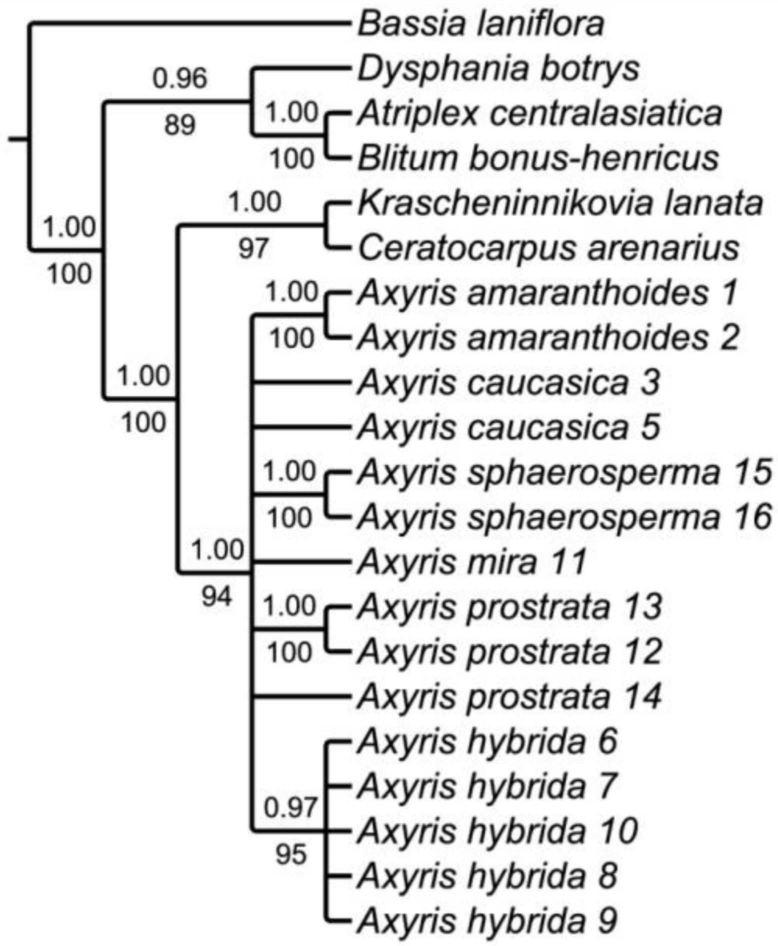

Figure S1. The ML phylogenetic cladogram of *Axyris* derived from nrITS. Posterior probabilities resulting from the Bayesian analysis are indicated above branches (only values ≥0.9); the numbers below branches refer to bootstrap values resulting from the ML analysis (only values ≥70).

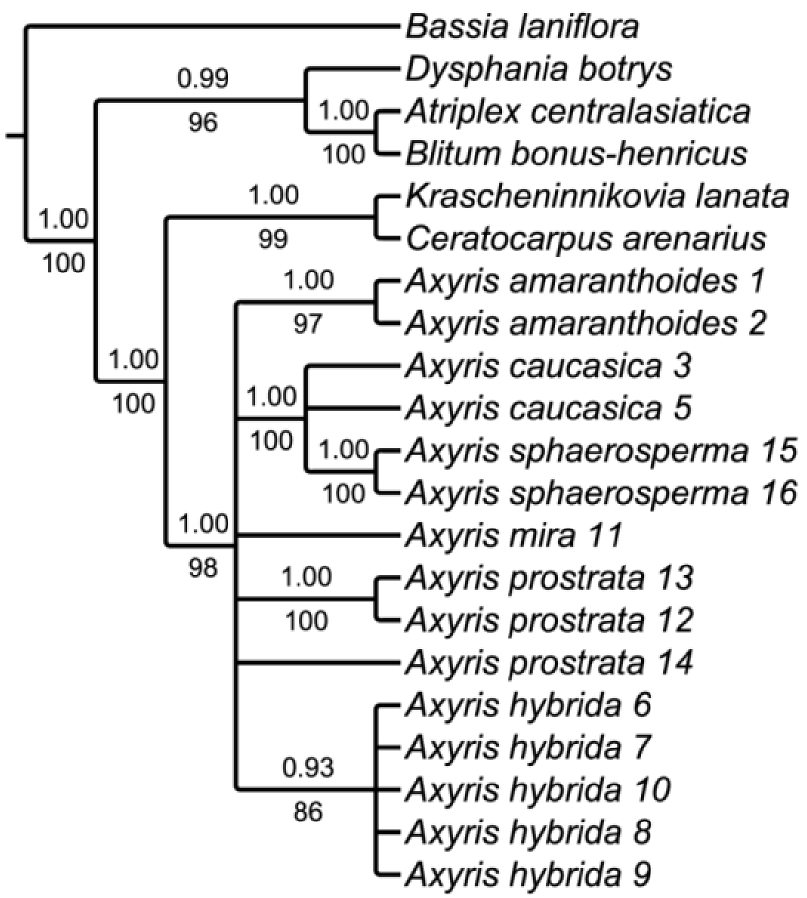

Figure S2. The ML phylogenetic cladogram of *Axyris* derived from the combined plastid matrix (rbcL, atpB-rbcL, and trnL-F). Posterior probabilities resulting from the Bayesian analysis are indicated above branches (only values ≥0.9), and the numbers below branches denote bootstrap values resulting from the ML analysis (only values ≥70).
